# Human Activity Dampens the Benefits of Group Size on Vigilance in Khulan (*Equus hemionus*) in Western China

**DOI:** 10.1371/journal.pone.0146725

**Published:** 2016-01-12

**Authors:** Mu-Yang Wang, Kathreen E. Ruckstuhl, Wen-Xuan Xu, David Blank, Wei-Kang Yang

**Affiliations:** 1 Key Laboratory of Biogeography and Bioresources in Arid Land, Xinjiang Institute of Ecology and Geography, Chinese Academy of Sciences, 830011 Urumqi, Xinjiang, China; 2 Department of Biological Sciences, University of Calgary, 2500 University Drive Northwest, Calgary, AB T2N 1N4, Canada; Department of Zoology, University of Cambridge, Downing Street, Cambridge CB2 3EJ, United Kingdom; Centre for Ecological and Evolutionary Studies, NETHERLANDS

## Abstract

Animals receive anti-predator benefits from social behavior. As part of a group, individuals spend less time being vigilant, and vigilance decreases with increasing group size. This phenomenon, called “the many-eyes effect”, together with the “encounter dilution effect”, is considered among the most important factors determining individual vigilance behavior. However, in addition to group size, other social and environmental factors also influence the degree of vigilance, including disturbance from human activities. In our study, we examined vigilance behavior of Khulans (*Equus hemionus*) in the Xinjiang Province in western China to test whether and how human disturbance and group size affect vigilance. According to our results, Khulan showed a negative correlation between group size and the percentage time spent vigilant, although this negative correlation depended on the groups’ disturbance level. Khulan in the more disturbed area had a dampened benefit from increases in group size, compared to those in the undisturbed core areas. Provision of continuous areas of high-quality habitat for Khulans will allow them to form larger undisturbed aggregations and to gain foraging benefits through reduced individual vigilance, as well as anti-predator benefits through increased probability of predator detection.

## Introduction

Animals often scan their surroundings when foraging to assess the potential predation risk. For animals living in groups, one major advantage is the decrease in the allocation of time dedicated to vigilance due to the many-eyes effect [1] and the encounter or risk-dilution effect [2,3]. As a result, individuals in larger groups can spend more time on other fitness-enhancing activities such as foraging [4]. This phenomenon is also called “the group size effect” [5].

Predation risk is a major selection pressure that determines the vigilance behavior of animals [6,7]. Research on human disturbance has begun to consider nonlethal disturbance from human activities as analogous to predation risk [8,9]. This 'human-caused predation risk' hypothesis states that animals also perceive disturbance from activities such as human recreation as a predation risk [10]. Since human disturbance can affect vigilance, researchers have studied this specific interaction in more detail [11–13]. While some studies found no changes in vigilance in more disturbed areas for some species [14], others found an effect [15,16]. In general, more studies show an increase in vigilance or an increase in group size when animals face high human disturbance level [17–20].

Recently, more studies focus on the combined effects of group size and human disturbance on vigilance [20]. For example, in highly disturbed areas, individual vigilance levels increased in bigger groups, causing the vigilance vs. group size curve to flatten, i.e., vigilance was high for all group sizes [21]. Hence, in disturbed areas a decrease in vigilance with group size would be countered by an increase in vigilance due to disturbance, and hence diminish the benefits of being in larger groups. In the present paper, we examine changes in time allocation to vigilance as a function of group size with two different disturbance levels. Khulans are listed in Appendix I of CITES, and classified as Endangered (EN) by IUCN [22]. While we predict that group size still does have an effect on individual vigilance levels, vigilance levels of individuals should be higher, and “the group size” effect should be weaker in intensely disturbed areas (buffer zone) than in less disturbed areas (core zone).

## Materials and Methods

### Study Area

**Fig 1:**
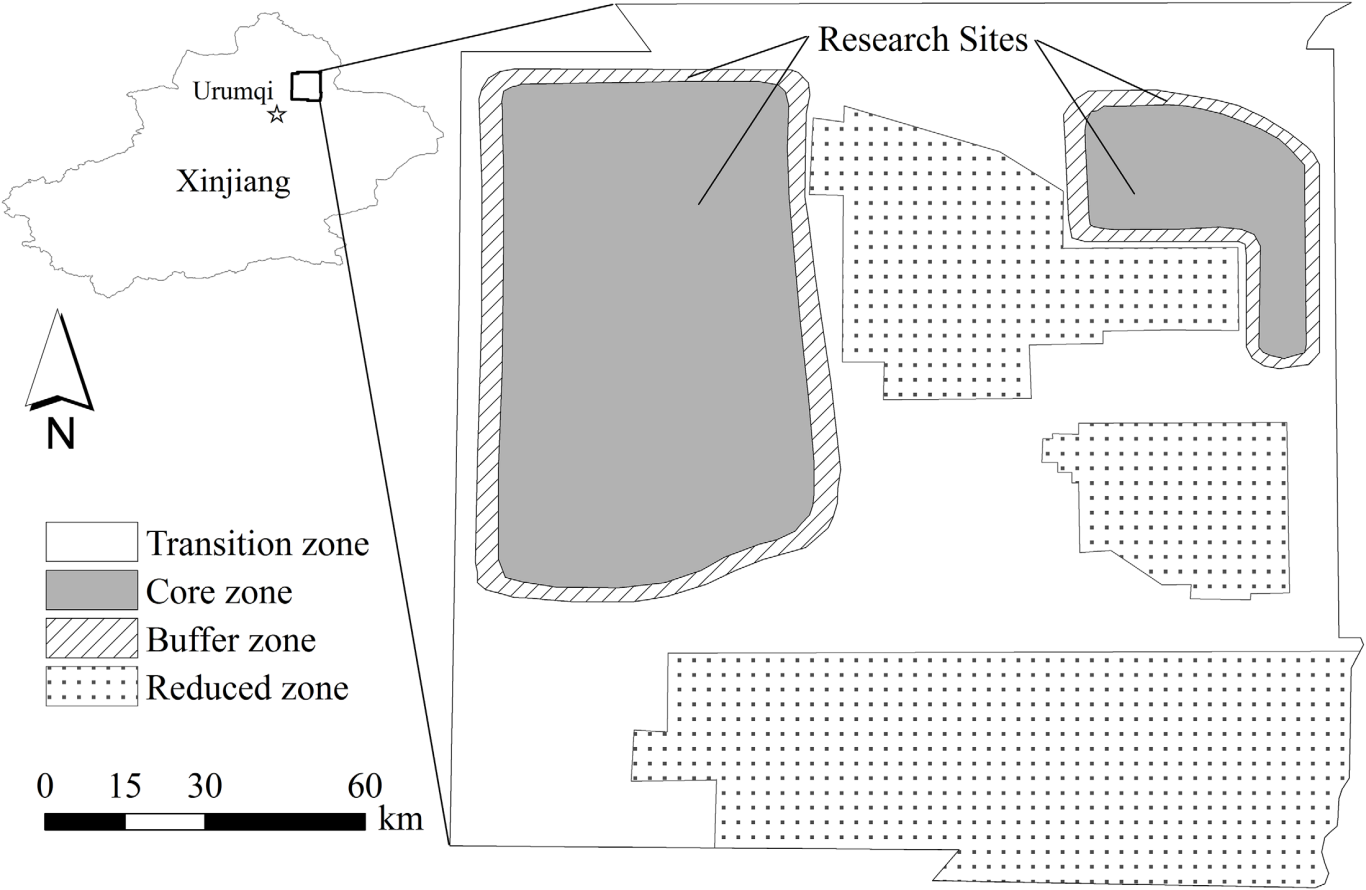
Location and functional zones of Kalamaili Nature Reserve.

This study was conducted in the Kalamaili Mountain Ungulate Nature Reserve (KNR) (44°36′-46°00′N, 88°30′-90°03′E), located in the eastern part of Junggar Basin, Xinjiang, China (Fig 1). The KNR is at an elevation of 600–1,700 m above sea level. This region has a harsh continental climate with an average yearly temperature of + 1.99°C. Winters are long and cold; summers are hot and short. Average annual rainfall is around 186.8 mm. Vegetation cover is quite sparse and consists mostly of desert shrubs and dwarf shrubs from the families Chenopodiaceae, Ephedraceae, Tamaricaceae, and Zygophyllaceae. The most common desert tree is the saxaul *Haloxylon ammodendron*. Common shrubs are *Anabasis salsa*, *Atraphaxis frutescens*, *Calligonum mongolicum*, *Ceratocarpus arenarius*, *Ceratoides latens*. Species from the genera *Sterigmostemum*, *Alyssum*, *Scorzonera* are common. Around 3,379–5,318 Khulans live in KNR [23]. The main predator of Khulans in the reserve are wolves, but the number of wolves is very low. Hence natural predation risk is very low and there is no recorded predation of Khulan by wolves in the two study areas.

In 2002 and 2004, KNR failed to upgrade its protection level due to the ongoing human activities inside the core reserve areas. Since 2005, KNR has been reduced from 18 000 km^2^ to 12 800 km^2^ due to coal mining. The KNR was divided into three zones in order to integrate biodiversity conservation and economic development. The total area of the core and buffer zones sharply decreased, which resulted in habitat fragmentation for wildlife [24]. Moreover, national road Nr. 216 is an important transportation route between Urumqi and Altay, and divides KNR into two parts. This study was conducted in two areas of the reserve: the buffer zone, which represents high human disturbance and the core zone which represents low human disturbance (Fig 1).

### Behavioral Sampling

The behavior of Khulans was investigated using the focal animal sampling method [25] during daylight hours in September 2014. Target groups were randomly selected, and observed using binoculars (magnification 8 ×) and a telescope (magnification 20 × 60) for distance viewing.

A group was defined as 2 or more individuals being in the same location, and inter-individual distances not exceeding 50 meters (the distance between individuals was visually estimated in Khulan body lengths). 50 meters is a fairly large distance and nearest neighbor distances were typically much closer. Individuals also had to show coordinated movements during an observation period to be considered as group members, which rules out animals >50m apart [26,27]. Group sizes can range from 2 to 270 individuals, but the group used in present paper were smaller than 23 with a median of 9. We defined vigilance behavior as when a feeding individual rapidly jerked its head up above shoulder length and scanned the environment. An individual was not considered to be vigilant when they were bedded. We took focal samples from as many groups as possible, with only a few individuals from the same group being observed. In this way, we tried to reduce the possibility of pseudo-replication. We were unable to distinguish between males and females, and age classes due to long observation distances. We divided the whole day into two parts: feeding peaks (8:00–10:00 and 18:00–20:00) vs. non-peaks according to Xia et al. (2013) [28]. For each sighting, the date, time of day, group size, and location (GPS) were recorded.

Each Khulan was observed for 10 minutes (min). Samples that lasted less than 10 min because of a subject leaving the group or the group disappeared were excluded from our analysis. We recorded all behaviors of focal animals using Aigo voice recorders to ensure the accuracy of the behavioral data. All data were transformed to digital files through the replaying of the audio recordings.

### Data Analysis

Percentage time vigilant was determined for each individual sampled and then the mean of all observed individual behaviors was calculated for the two different study areas. Mean percent time spent vigilant was used as the dependent variable and time of day and group ID as predictor variable. Vigilance rate represented the number of vigilance bouts per min of observation. Vigilance duration was the length of time measured in seconds (s) for a single vigilance act. By using only one value (mean) per group this avoided pseudo-replication. To normalize distributions, we used the arcsine square-root transformation for time spent vigilant and vigilance rate, and the logarithmic base 10 transformation for vigilance duration. We conducted a General Linear Model (GLM) including area (core vs. buffer) and time of day (feeding peak and non-peak) as fixed factor, and group size as a covariant to test the effect of group size, time of day, and disturbance level on vigilance behavior. Statistical analyses were done using R software (version 3.1.3). Significance levels were set at 0.05.

### Ethics Statement

Altay Forestry Bureau permitted us to conduct this work in the Kalamaili Mountain Ungulate Nature Reserve. No further approval by an Ethics Committee was required, as behavioral observations at a distance were non-invasive.

## Results

We obtained 532 focal observations, representing a total of 5320 min of observation. A total of 257 focal observations (48%) were from the core zone while 275 samples (52%) were obtained from the buffer area. Group size was 10.25 (±3.98SE), ranging from 3 to 20 in the core zone, and had an average of 12.35 (±6.36SE) ranging from 2 to 22 in the buffer zone.

**Fig 2:**
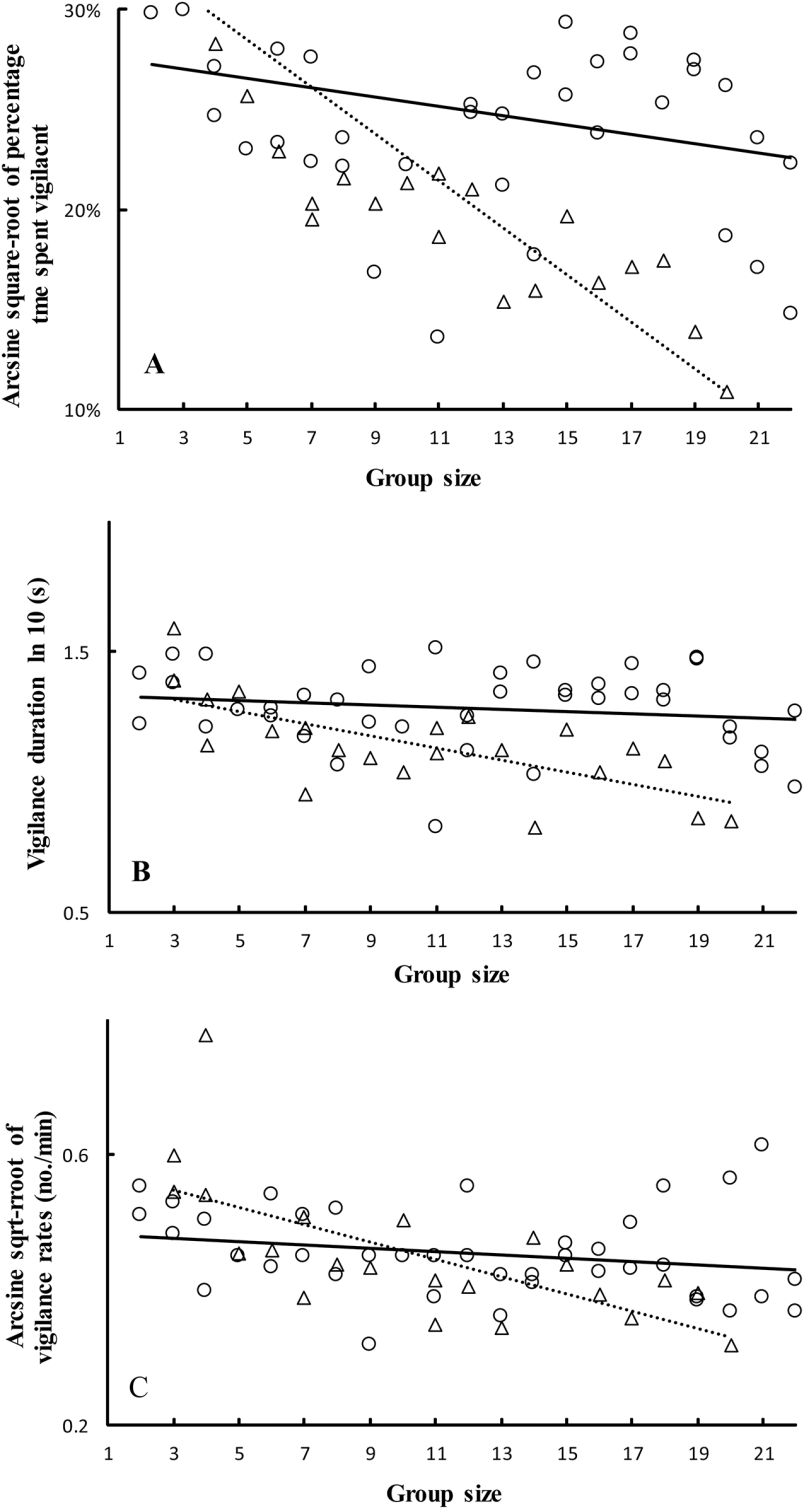
Percentage time spent vigilant (A), vigilance rate (B), and vigilance duration (C) as a function of group size and disturbance level areas for Khulan in Kalamaili Nature Reserve (KNR), Xinjiang, China, using transformed data. The average value of each group at various sizes and under different circumstance is used for each vigilance variable. The dashed line is the predicted line of group size in the core zone (triangles). The solid line is the predicted line of group size in the buffer zone (circles).

Khulan spent 4.81% (±0.27%SE; range: 0.33% - 22.83%) of their time being vigilant, in the low disturbance core zone, and 6.74% (±0.23%SE; range: 0.50% - 20.67%) in the high disturbance buffer zone. They thus spent more time being vigilant in the buffer zone than core zone (Table 1). Time of day had no significant influence on percentage time spent vigilant (Table 1). There was a clear decrease in percentage time spent vigilant with group size- but there was a significantly different extent of decrease in the two areas as revealed by a significant interaction between area and group size (Table 1, Fig 2A).

**Table 1:** Results of General Linear Models testing differences in percentage time spent vigilant, vigilance duration, and vigilance rates, with group size, and disturbance level. The percentage time spent vigilant was highly significant (F_4,57_ = 11.66, P <0.001, adjusted R^2^ = 0.412) as was vigilance duration (F_4,57_ = 7.097, P <0.001, adjusted R^2^ = 0.286). The vigilance rates model was also significant (F_4,57_ = 5.522, P <0.001, adjusted R^2^ = 0.229). The intercept for all three models was the core zone.

			Percentage time spent vigilant			Vigilance duration			Vigilance rates	
Source	df	t	p	Est.	t	p	Est.	t	p	Est.
Intercept	1	13.636	< 0.001	0.344 ± 0.025	17.884	< 0.001	3.155 ± 0.176	15.414	< 0.001	0.620 ± 0.040
Group size	1	-5.789	< 0.001	-0.012 ± 0.002	-3.583	< 0.001	-0.051 ± 0.014	-4.398	< 0.001	-0.014 ± 0.003
Area	1	-2.319	0.024	-0.068 ± 0.029	-0.529	0.599	-0.109 ± 0.206	-2.514	0.015	-0.118 ± 0.047
Time of day	1	0.751	0.455	0.010 ± 0.013	0.472	0.638	0.043 ± 0.092	-0.008	0.994	-0.0002 ± 0.02
Group size * area	4	3.930	< 0.001	0.009 ± 0.002	2.491	0.016	0.042 ± 0.017	3.044	0.004	0.012 ± 0.004

Mean vigilance bout duration was 14.748 s (±0.697SE) and ranged from 2 to 70 s in the core zone, while mean bout duration was 21.38 s (±0.598SE) and ranged from 3 to 60 s in the buffer zone. Khulans showed the same vigilance bout duration in the two areas, and during different times of day (Table 1). There was a clear decrease in bout duration with group size- but a significantly different extent of decrease in the two areas as revealed by a significant interaction between area and group size (Table 1, Fig 2B).

Mean vigilance rate was 0.201 scans/min (±0.008SE), ranging from 0.1 to 0.9 in the core zone, and 0.211 scans/min (±0.006), and ranging from 0.1 to 0.8 in the buffer zone. Again there was a significant effect of area, group size and a significant interaction between group size and area on vigilance rate (Table 1, Fig 2C).

## Discussion

Percent time spent vigilant, vigilance bout duration, and vigilance rates in Khulan were all significantly affected by group size but an interaction between group size and area revealed that the effect of group size differed between the two areas for all dependent variables. Previous studies have confirmed that the level of disturbance caused by human activities influences the allocation of time to vigilance in some species [29–31]. However, in this study we showed that the level of disturbance also influences levels of vigilance in Khulan when found in different areas (buffer vs. core). The quality and abundance of food in KNR in Xinjiang, China did not differ between the two observation areas. In general, the core zone of the reserve was built for the protection of wildlife and there was restricted access compared with the buffer zone. In our study area, both areas were reduced six-fold due to development for energy exploitation. Increased fragmentation of habitat is bringing humans in closer contact with wild populations. Such higher nuisance disturbances may force Khulans into larger groups, and to devote more time to safety-related behaviors, such as increased levels of vigilance [32]. Therefore, it is important to provide continuous and large areas of habitat that can support bigger groups of Khulans. Bigger groups result in benefits through reduced individual vigilance and increased anti-predation benefits through increased probability of predator detection.

It is important for conservation efforts to understand the interaction effects between human nuisance disturbance and group size on animal behavior. For example, group size and human disturbance were shown to have combined effects on vigilance behavior of mountain gazelle (*Gazella gazella*) [21]. The study concluded that the group size effect on vigilance decreased when human disturbance increased. That is, the vigilance levels remained unchanged no matter what their group size and intensity of human disturbance [20].

Our study found similar results on the interaction between human disturbance and group size. The results indicated that the group size effect breaks down when there is increased disturbance in the buffer zones. More time devoted to vigilance in bigger groups when they stay in high disturbance areas counteracts the benefits of the group size effect. Individuals, therefore, could not gain any benefits from the formation of larger groups (i.e. from reduced vigilance). As a consequence, reducing disturbance is important for Khulans because it allows the group size effect to operate normally.

### Conclusion

In conclusion, we found that increases in group size of Khulans reduced the time they devoted to vigilance. However, human disturbance led to a higher vigilance level and a breakdown of the potential foraging benefits of the group-size effect on vigilance. Recently, it was confirmed that human interference would also dramatically affect water utilization by Khulan in KNR [33]. Therefore, the implications for conservation of the Khulan in our study area are to maintain continuous areas of high-quality habitat, which are more likely to allow for the formation of larger undisturbed aggregations that can probably benefit from the group-size effect on vigilance.

## Supporting Information

S1 Table Percentage time spent vigilant, vigilance rate, and vigilance duration as a function of group size, disturbance level areas and time of day of Khulan in Kalamaili Nature Reserve (KNR). (XLSX)
